# Subcortical gray matter volumes and 5‐year dementia risk in individuals with subjective cognitive decline or mild cognitive impairment: A multi‐cohort analysis

**DOI:** 10.1002/alz.70413

**Published:** 2025-07-08

**Authors:** Mathijs T. Rosbergen, Pieter van der Veere, Jacqueline J. Claus, Tavia E. Evans, Vikram Venkatraghavan, Frederik Barkhof, Argonde C. van Harten, M. Arfan Ikram, Wiesje M. van der Flier, Meike W. Vernooij, Frank J. Wolters

**Affiliations:** ^1^ Department of Epidemiology Erasmus MC – University Medical Center Rotterdam Rotterdam the Netherlands; ^2^ Department of Radiology & Nuclear Medicine and Alzheimer Centre Erasmus MC – University Medical Center Rotterdam Rotterdam the Netherlands; ^3^ Amsterdam Neuroscience, Neurodegeneration Amsterdam Zuidoost the Netherlands; ^4^ Department of Epidemiology and Data Science Amsterdam UMC location Vrije Universiteit Amsterdam Amsterdam the Netherlands; ^5^ Alzheimer Center Amsterdam, Neurology Vrije Universiteit Amsterdam, Amsterdam UMC location VUMC Amsterdam the Netherlands; ^6^ Department of Clinical Genetics Erasmus MC – University Medical Center Rotterdam the Netherlands; ^7^ Department of Radiology and Nuclear Medicine Amsterdam Neuroscience, Vrije Universiteit Amsterdam Amsterdam the Netherlands; ^8^ Dementia Research Centre, UCL Queen Square Institute of Neurology University College London, National Hospital for Neurology and Neurosurgery London UK

**Keywords:** Alzheimer's disease, dementia, magnetic resonance imaging, neuroimaging, subcortical structures

## Abstract

**INTRODUCTION:**

The prognostic value of subcortical gray matter structures for dementia beyond the hippocampus remains unclear.

**METHODS:**

We included participants with subjective cognitive decline or mild cognitive impairment from two memory clinic‐based cohorts (Amsterdam Dementia Cohort and National Alzheimer's Coordinating Center) and one population‐based cohort (Rotterdam Study). We assessed volumes of subcortical structures on magnetic resonance imaging and determined 5‐year dementia risk using Cox models.

**RESULTS:**

Of 7076 participants (mean age: 66–69 years, 58.8%–61.0% women; *N*_SCC_ = 5425, *N*_MCI_ = 1661), 622 developed dementia within 5 years. Smaller volumes of the hippocampus and amygdala were consistently associated with increased dementia risk, independent of other subcortical structures. Smaller hippocampal volume was predominantly associated with the clinical diagnosis of Alzheimer's disease, but the prognostic value did not differ by amyloid status.

**DISCUSSION:**

Hippocampal and amygdalar volume are consistently associated with dementia risk in individuals with subjective cognitive decline or mild cognitive impairment, which may hold potential for personalized prognosis.

**Highlights:**

- Seven thousand seventy‐six participants from three large longitudinal cohorts were followed for a maximum of 5 years.
- Hippocampal volume is associated with 5‐year risk of dementia in subjective cognitive decline (SCD) or mild cognitive impairment (MCI).
- Amygdalar volume is associated with a 5‐year risk of dementia in SCD or MCI.
- Stratifying by SCD and MCI revealed no consistent major differences.

## 1 BACKGROUND

Dementia is one of the leading causes of disability and dependency worldwide, with 55 million people living with dementia globally.^1^ Diagnosis primarily relies on clinical symptoms but is often supported by neuroimaging to differentiate from non‐degenerative causes of cognitive impairment and characterize disease etiology. For example, atrophy of the medial temporal lobe and the hippocampus are commonly observed with Alzheimer's disease (AD),^2^ and widely acknowledged as an important marker of AD pathology in clinical diagnosis. Applied to prognosis for people with subjective cognitive decline (SCD)^3^ or mild cognitive impairment (MCI), such markers could tailor follow‐up strategies and care planning, as well as inclusion in trials of disease‐modifying therapy. Yet, the significance of imaging measures for determining prognosis is uncertain, depending on reliable assessment methods and sufficient discriminatory value that is unlikely achieved by any single imaging marker on its own.^4^ The increasing application of volumetric brain assessment in clinical practice offers new possibilities for improving prognostic information to patients. In particular, volumetric assessment allows for the assessment of subcortical gray matter structures that cannot be measured through visual rating scales, taking risk assessment beyond the hippocampus alone.

Prior neuroimaging studies have suggested that various subcortical brain structures play a key role in dementia pathophysiology.^5^ These gray matter structures, which are not part of the neocortex, include the nucleus accumbens, amygdala, caudate nucleus, hippocampus, globus pallidum, putamen, and thalamus. They are highly connected to each other and cortical gray matter and involved in a wide range of functions, including cognition, emotion, autonomic control, and language,^6^ all of which are often affected in individuals with dementia. Despite their functional relevance, few longitudinal studies have assessed the prognostic value of subcortical brain structure volumes in dementia risk beyond the hippocampus. Evidence from these studies is inconsistent,^7^, ^8^, ^9^, ^10^ which could be attributable to differences in studied populations, disease stage, follow‐up, and brain imaging. Moreover, the role of subcortical brain structures may differ between AD and other causes of dementia, depending on the underlying dementia etiology.^11^, ^12^, ^13^ Therefore, larger studies covering a wide range of the neurodegenerative disease spectrum are needed to determine the role of early neurodegenerative changes within subcortical gray matter structures in risk assessment for different causes of dementia.

In this study, we aimed to determine the prognostic value of several subcortical gray matter volumes for progression to dementia in individuals with SCD or MCI. Specifically, we assessed volumes of the nucleus accumbens, amygdala, caudate nucleus, hippocampus, globus pallidum, putamen, and thalamus in two clinic‐based cohorts and one population‐based cohort from the United States and Europe.

## 2 METHODS

### 2.1 Study population

We included participants with SCD and MCI from two memory clinic‐based cohorts (Amsterdam Dementia Cohort [ADC] and the Alzheimer's Disease Research Centers of National Alzheimer's Coordinating Center [NACC], funded by National Institute on Aging (NIA)/National Institutes of Health Grant U24 AG072122) and one population‐based cohort (the Rotterdam Study). Details of the design and characteristics of participating studies have been described previously^14^, ^15^, ^16^ and are summarized below. All studies were approved by the relevant institutional review boards, and written informed consent was obtained from all participants.

#### 2.1.1 Amsterdam dementia cohort

Since the year 2000, all patients attending the Alzheimer Center Amsterdam of Amsterdam University Medical Center, in Amsterdam, are asked to participate in the ADC and consent for their clinical data to be used for scientific purposes. Patients undergo a standardized 1‐day screening battery, including neuropsychological evaluation, brain magnetic resonance imaging (MRI), and cerebrospinal fluid (CSF) sampling.^15^ Participants with SCD are invited for annual follow‐up visits in the context of the Subjective Cognitive Impairment Cohort (SCIENCe) project,^17^ while MCI patients are invited for clinical follow‐up visits at which their clinic status is reassessed. The current study includes all participants with available MRI data and who were diagnosed with SCD or MCI between 2001 and 2022.

#### 2.1.2 NACC

NACC includes data from 41 past and current NIA‐supported Alzheimer's Disease Centers across the United States. Data from these centers have been collected since 2005 into a large database of standardized clinical and neuropathological research data, including data on brain MRI if available. Follow‐up data were collected annually during in‐person visits and telephone calls. For the current study, we included all participants who were labeled as normal cognition, SCD, or MCI and underwent brain MRI between 2005 and 2022.

#### 2.1.3 Rotterdam Study

The Rotterdam Study is an ongoing, population‐based cohort study in the city of Rotterdam, the Netherlands. Residents aged ≥ 40 years living in the Ommoord district were invited to participate across four recruitment waves from 1990 onward. Participants were examined extensively at the study research center every 5 years, including routine cognitive assessment and brain MRI from 2005 onward.^14^, ^18^ For the present study, we included participants with SCD or MCI who underwent brain MRI between 2005 and 2015.

### 2.2 MRI acquisition and processing

MRI in the ADC was performed on site using 1T and 1.5T scanners before 2008 (Magnetom Avanto, Impact, and Sonata, Siemens; Signa, GE Healthcare) and using 3T scanners from 2008 onward (Magnetom Siemens; Discovery MR750, Signa General Electric Medical Systems; Ingenuity TF PET/MR, Philips Medical Systems; and Titan, Toshiba Medical Systems). All scans within this cohort were performed using a standardized imaging protocol.^15^ MRI scans in NACC were performed with 1.5T and 3.0T scanners (General Electric Healthcare, Siemens and Philips). Different scan protocols were used among different research centers. MRI scans in the Rotterdam Study were performed on the same 1.5T scanner (Signa Excite II, General Electric Healthcare) throughout the study duration. The scan protocol and sequence details of the Rotterdam Study have been previously described elsewhere.^18^

To obtain volumetric measurements, T1‐weighted MRI scans were processed using FreeSurfer. MRI scans of ADC were processed with FreeSurfer 7.0, while MRI scans of NACC and the Rotterdam Study were processed using FreeSurfer 6.0. For the current study, we used volumetric measurements of nucleus accumbens, amygdala, caudate nucleus, hippocampus, globus pallidum, putamen, thalamus, cortical gray matter, and intracranial volume.

**RESEARCH IN CONTEXT**

1. **Systematic review**: The authors reviewed the literature on subcortical gray matter structures in dementia. Previous studies have largely focused on the hippocampus as a key marker for dementia risk, especially in Alzheimer's disease. While the hippocampus has been widely studied, the role of other subcortical structures has been less explored, with mixed results across cohorts.
2. **Interpretation**: Our findings demonstrate that both hippocampal and amygdalar volumes are consistently associated with dementia risk in individuals with subjective cognitive decline or mild cognitive impairment, independently of other subcortical volumes.
3. **Future directions**: Future research using volumetrics of subcortical structures should go on beyond the hippocampus alone, to further evaluate the joint role of the amygdala and hippocampus for dementia prognosis and prediction.

### 2.3 Diagnostic assessment and follow‐up for dementia

Within the ADC, diagnoses of dementia and MCI were made in a multidisciplinary consensus meeting.^15^ Diagnosis of all‐cause dementia was entirely based on clinical evaluation, while subtyping the specific etiologies, such as AD, is supported by biomarker data, including hippocampal atrophy. All diagnoses fulfilled the core clinical criteria NIA–Alzheimer's Association criteria.^19^, ^20^ Patients with MCI fulfilled the criteria defined by Petersen.^19^, ^21^ When clinical investigations showed no impairment (i.e., criteria for MCI, dementia, or any psychiatric disorder were not met), patients were considered to have a SCD diagnosis and were reassessed in person during annual follow‐up visits. The diagnostic procedure **within NACC varies** between centers, from consensus panel diagnosis to a single physician's assessment, all adhering to standardized clinical criteria as outlined by the Diagnostic and Statistical Manual of Mental Disorders Fourth Edition (DSM‐IV) and National Institute of Neurological and Communicative Disorders and Stroke and the Alzheimer's Disease and Related Disorders Association (NINCDS‐ADRDA). Diagnosis of MCI was made predominantly via a consensus conference using modified Petersen criteria.^21^ Diagnoses status were re‐evaluated during annual data collection. In the Rotterdam Study, participants were screened for dementia at baseline and during follow‐up examinations every 5 years using the Mini‐Mental State Examination (MMSE) and the Geriatric Mental Schedule (GMS) organic level. All participants underwent a brief neuropsychological examination,^22^ with additional CAMDEX and informant interview upon indication. A consensus panel, led by a consultant neurologist, decided on the final dementia diagnosis in all cases, in accordance with the DSM‐III‐R criteria for dementia and the NINCDS‐ADRDA criteria for clinical AD dementia. All participants were also continuously monitored for diagnosis of dementia through continuous linkage to electronic medical records. Classification of SCD and MCI was based on interview questions and cognitive assessment during research center visits. During interviews, three questions regarding memory decline (difficulty remembering, forgetting what one had planned to do, and difficulty finding words) were asked. One confirmative answer to any of these questions was considered a SCD. MCI was defined using criteria based on the criteria by Petersen^23^ and a detailed description of the MCI assessment in the Rotterdam Study has been published previously.^24^

Across cohorts, volumetric measures were not used for baseline diagnosis in this sample. Although diagnosis of dementia was entirely based on clinical evaluation, neuroradiologist assessment of global atrophy and medial temporal lobe atrophy were part of routine work‐up to determine etiologic diagnoses, including AD, in the ADC and NACC.

### 2.4 Assessment of educational attainment and amyloid status

In the ADC, data on educational attainment were obtained during baseline visit using the Verhage scale.^25^ To enhance comparability of characteristics in Table 1, the seven categories of the Verhage scale were transformed into four groups similar to the education categories of the Rotterdam Study. Educational attainment within NACC was measured at baseline visits as total years of education. Transforming this measurement into education categories was unfortunately not possible. Information regarding educational attainment in the Rotterdam Study was obtained by trained interviewers during baseline home interviews and categorized into four groups, ranging from primary to higher education (higher vocational education or university). In the ADC, data on amyloid positivity in a subset of participants were obtained through CSF or amyloid positron emission tomography (PET). Before 2018, sandwich enzyme‐linked immunosorbent assays (ELISA) were used (Innotest, Fujirebio). From 2018 onward, CSF was analyzed using Elecsys (Roche). Amyloid PET imaging was performed using 3‐Tesla Ingenuity TF PET/MRI, Ingenuity TF PET/CT (computed tomography) and Gemini TF PET/CT scanners (Philips Healthcare) with the 11C‐Pittsburg compound B (PiB), 18F‐flutemetamol, and 18F‐Florbetaben compounds.^26^, ^27^, ^28^ Participant were labelled amyloid positive when either their CSF or amyloid PET was positive.

**TABLE 1:** Demographic and imaging characteristics for the Amsterdam Dementia Cohort, National Alzheimer's Coordinating Center cohort, and the Rotterdam Study.

	Amsterdam Dementia Cohort (*N* = 1964)	NACC (*N* = 2207)	Rotterdam Study (*N* = 2905)
Age, years	61.9 [9.1]	68.9 [11.0]	66.1 [11.0]
Sex, female	1170 [59.6%]	1347 [61.0%]	1708 [58.8%]
Education duration, years	n.a.	15.8 [6.0]	n.a.
Education			
Primary only	20 [1.0%]	n.a.	283 [9.7%]
Lower vocational	154 [7.8%]	n.a.	1147 [39.5%]
Intermediate vocational/higher general	830 [42.3%]	n.a.	877 [30.2%]
Higher vocational/university	958 [48.8%]	n.a.	598 [20.6%]
Subjective cognitive decline	1254 [63.8%]	1485 [67.4%]	2686 [92.1%]
Mild cognitive impairment	710 [36.2%]	722 [32.6%]	229 [7.9%]
MRI field strength			
0.95 Tesla	340 [17.3%]	0	0
1.5 Tesla	212 [10.8%]	774 [35.1%]	2905 [100.0%]
3.0 Tesla	1412 [71.9%]	1,428 [64.7%]	0
Unspecified	0	5 [0.2%]	0
Brain structure volumes			
Accumbens volume, mL	0.47 [0.11]	0.44 [0.11]	0.49 [0.09]
Amygdala volume, mL	1.54 [0.27]	1.46 [0.26]	1.42 [0.20]
Caudate volume, mL	3.40 [0.54]	3.29 [0.52]	3.31 [0.51]
Hippocampus volume, mL	3.85 [0.52]	3.65 [0.50]	3.84 [0.42]
Pallidum volume, mL	1.88 [0.25]	1.77 [0.25]	1.57 [0.22]
Putamen volume, mL	4.48 [0.62]	4.19 [0.62]	4.13 [0.56]
Thalamus volume, mL	6.79 [0.85]	6.42 [0.77]	6.48 [0.71]
Cortical gray matter volume, mL	543.20 [63.0]	528.77 [58.09]	536.30 [50.25]
Amyloid status			
Negative	827 [68.1%]	–	–
Positive	387 [31.9%]	–	–

*Note*: Continuous variables are presented as means [standard deviations] and categorical variables as numbers (percentages).

Abbreviations: mL, milliliter; MRI, magnetic resonance imaging; NACC, National Alzheimer's Coordinating Center.

### 2.5 Data analysis

We imputed missing data on covariables separately for each cohort. In the ADC and the Rotterdam Study, data for educational attainment were missing for 0.1% and 0.9%, respectively. Within NACC, some data were missing for MRI scanner and field strength (< 13.9%). For imputation, we used the mice package in R (version 4.3.3 and version 4.4.2.) to perform 5‐fold multiple imputation with the number of iterations set to default (*n* = 5). To assess the quality of the imputation, we examined the density plots, which showed that the distributions of variables were similar in the imputed and non‐imputed datasets. We standardized subcortical volumes to facilitate comparison across structures and provide hazard ratios (HRs) per standard deviation (SD) increase of the volume of each subcortical structure. For bilateral structures, we used the mean of both values for analyses. Follow‐up time was measured in years from the date of MRI until dementia diagnosis, death, loss to follow‐up, or censoring at 5 years of follow‐up, whichever came first. Information on death was only used for data from the Rotterdam Study; for the ADC and NACC, censoring occurred at the time of the last clinical assessment.

Primary analysis consisted of Cox proportional hazard models to obtain HRs and 95% confidence intervals (CIs) for the association between subcortical volumes and risk of dementia in each cohort. For all Cox models, we tested the proportional hazard assumption. In secondary analyses, we stratified (1) between AD and other causes of dementia as an outcome, (2) by baseline amyloid positivity in the subsample of the ADC with CSF or amyloid PET, and (3) by baseline diagnosis of SCD versus MCI.

After the primary analysis, we performed a post hoc analysis in which we compared the C‐statistic between different models: a basic model including age, sex, and education; the basic model including hippocampal volume as an additional predictor; and the basic model including hippocampal volume and amygdalar volume as additional predictors. We performed this analysis to determine the improvement in prognostic value when using hippocampal volume and both hippocampal and amygdalar volume. Models including volumetric measurements were additionally adjusted for different MRI scanners, field strengths, and intracranial volume.

We further did sensitivity analyses, (1) stratifying by sex, while testing for multiplicative interactions between the volume of each subcortical structure and sex; (2) repeating the primary analysis on a subsample from NACC consisting of participants who visited the research center for clinical evaluation, excluding those who volunteered solely research participation; (3) repeating primary analysis on subsamples from NACC and the ADC consisting of participants who underwent brain MRI with a field strength of 3.0T; and (4) repeating primary analysis with adjustment for different MRI scanners and field strengths by applying neuroCombat harmonization on the extracted volumes instead of adjustment through covariates. The second sensitivity analysis was performed to select within the NACC population on a clinical sample, similar to the ADC. All of these sensitivity analyses included participants classified as SCD as well as participants classified as MCI.

For all analyses, we constructed three different models: adjusting for intracranial volume, MRI field strength and MRI scanner (model 1); adjusting for intracranial volume, MRI field strength, MRI scanner age, sex, and education (model 2); and additionally adjusting for volumes of other subcortical structures and global cortical gray matter volume (model 3) to investigate if there is an independent effect. In the Rotterdam Study, we did not adjust for MRI field strength and MRI scanner, as all participants within this cohort were scanned with the same field strength and MRI scanner. We did not meta‐analyze risk estimates across studies, because we anticipated heterogeneity and intended to demonstrate associations in both clinic‐based and community‐based setting.

All analyses were performed in R (version 4.3.3 and version 4.4.2; packages: haven, dplyr, mice, tableone, survival, neuroCombat). Alpha was set at 0.05.

## 3 RESULTS

We included a total of 7076 participants with SCD or MCI of whom 1964 participants originated from the ADC, 2207 participants from NACC, and 2905 from the Rotterdam Study. Baseline characteristics for each cohort are presented in Table 1. Mean age at time of the MRI scan ranged from 61.9 to 68.9 years, and in each cohort ≈ 60% of participants were women. In the clinic‐based ADC and NACC, approximately one third of participants had MCI, compared to 8% in the population‐based Rotterdam Study.

During the maximum follow‐up time of 5 years, dementia was diagnosed in 701 (9.9%) individuals. Progression to dementia occurred in 249 (12.7% of cohort; *N*_SCD _= 31 [2.5% of SCD in cohort]; *N*_MCI _= 218 [30.7% of MCI in cohort]) individuals in ADC, 292 (13.2% of cohort; *N*_SCD _= 26 [1.8% of SCD in cohort]; *N*_MCI _= 266 [36.8% of MCI in cohort]) in NACC, and 81 (2.8% of cohort; *N*_SCD _= 62 [2.3% of SCD in cohort]; *N*_MCI _= 19 [8.3% of MCI in cohort]) in the Rotterdam Study. Dementia incidence per time point is shown in Table S1 in supporting information.

### 3.1 Subcortical structures and risk of dementia

**FIGURE 1:**
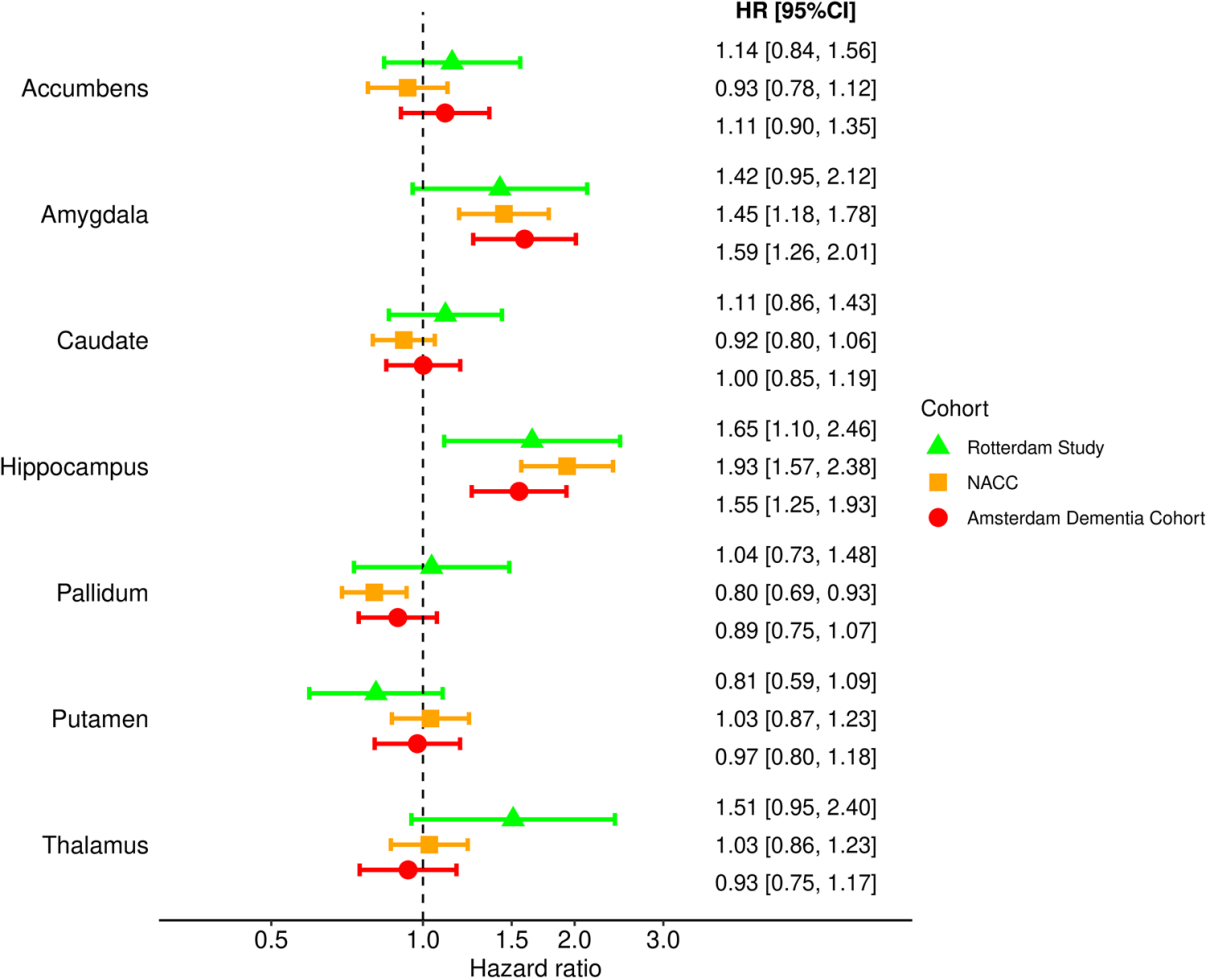
Subcortical structures and the risk of dementia. Hazard ratio per standard deviation decrease of subcortical structure volume, adjusted for age, sex, education, MRI field strength, MRI scanner, intracranial volume, cortical gray matter, and volumes of all other subcortical structures. Error bars depict 95% confidence intervals. CI, confidence interval; HR, hazard ratio; MRI, magnetic resonance imaging; NACC, National Alzheimer's Coordinating Center.

Results from the crude models and models adjusted for age, sex, and education are shown in Table 2. Consistently across cohorts, smaller hippocampal volume was significantly associated with an increased dementia risk (HR [95% CI] in the Rotterdam Study: 1.65 [1.10–2.46]; in NACC: 1.92 [1.56–2.37]; and in ADC: 1.55 [1.25–1.93]) in fully adjusted models (Figure 1, Table 2). Similarly, smaller amygdalar volume was associated with increased dementia risk in both clinic‐based cohorts (HR [95% CI] in NACC: 1.46 [1.19–1.79]; and in ADC: 1.59 [1.26–2.01]), with similar effect estimates in the population‐based cohort (HR [95% CI]: 1.42 [0.95–2.12]). Smaller volume of the pallidum was significantly associated with decreased dementia risk in NACC, but not in either of the other cohorts (Table 2). Smaller volume of the thalamus was not associated with dementia risk in the clinic‐based cohorts, while a moderate, not statistically significant increase was observed in the Rotterdam Study (HR [95% CI]: 1.51 [0.95–2.40]). None of the other structures showed an association with dementia risk in any of the studies. In a post hoc analysis, hippocampal volume improved the C‐statistic substantially across cohorts, compared to the basic models (Table S2 in supporting information). In both memory clinic cohorts, adding amygdalar volume on top of hippocampal volume again improved the C‐statistic, compared to the model with hippocampal volume alone.

**TABLE 2:** Subcortical structures and risk of dementia.

	Cohort	Model 1 HR [95% CI]	Model 2 HR [95% CI]	Model 3 HR [95% CI]
Accumbens	Rotterdam Study	2.57 [1.99–3.32]	1.42 [1.07–1.89]	1.14 [0.84–1.56]
	NACC	2.01 [1.75–2.31]	1.60 [1.37–1.87]	0.93 [0.78–1.12]
	ADC	1.89 [1.61–2.21]	1.59 [1.36–1.87]	1.11 [0.90–1.35]
Amygdala	Rotterdam Study	3.95 [3.07–5.08]	2.27 [1.69–3.05]	1.42 [0.95–2.12]
	NACC	2.70 [2.39–3.04]	2.49 [2.18–2.84]	1.46 [1.19–1.79]
	ADC	2.45 [2.11–2.83]	2.35 [2.01–2.74]	1.59 [1.26–2.01]
Caudate	Rotterdam Study	0.74 [0.59–0.91]	1.07 [0.85–1.33]	1.11 [0.86–1.43]
	NACC	1.09 [0.95–1.25]	1.10 [0.97–1.25]	0.92 [0.80–1.06]
	ADC	1.20 [1.02–1.41]	1.12 [0.97–1.29]	1.00 [0.85–1.19]
Hippocampus	Rotterdam Study	4.03 [3.20–5.07]	2.37 [1.76–3.18]	1.65 [1.10–2.46]
	NACC	2.96 [2.61–3.35]	2.71 [2.36–3.12]	1.92 [1.56–2.37]
	ADC	2.31 [2.01–2.65]	2.15 [1.87–2.48]	1.55 [1.25–1.93]
Pallidum	Rotterdam Study	2.22 [1.68–2.94]	1.27 [0.95–1.69]	1.04 [0.73–1.48]
	NACC	1.22 [1.05–1.38]	0.99 [0.87–1.14]	0.80 [0.69–0.93]
	ADC	1.19 [1.03–1.39]	1.10 [0.95–1.26]	0.89 [0.75–1.07]
Putamen	Rotterdam Study	2.02 [1.55–2.63]	1.13 [0.88–1.45]	0.81 [0.59–1.09]
	NACC	1.61 [1.41–1.83]	1.31 [1.14–1.51]	1.04 [0.87–1.24]
	ADC	1.46 [1.25–1.70]	1.27 [1.10–1.48]	0.97 [0.80–1.18]
Thalamus	Rotterdam Study	4.47 [3.19–6.27]	2.05 [1.37–3.06]	1.51 [0.95–2.40]
	NACC	2.09 [1.79–2.44]	1.62 [1.36–1.92]	1.02 [0.86–1.22]
	ADC	1.75 [1.48–2.08]	1.50 [1.26–1.79]	0.93 [0.75–1.17]

*Note*: Results from a Cox regression model for the Amsterdam Dementia Cohort, National Alzheimer's Coordinating Center cohort, and the Rotterdam Study. Model 1: adjusted for MRI scanner (settings) and intracranial volume.

Model 2: adjusted for MRI scanner (settings), intracranial volume, age, sex, and education.

Model 3: adjusted for MRI scanner (settings), intracranial volume, age, sex, education, other subcortical structure volumes, and cortical gray matter volume.

Abbreviation: ADC, Amsterdam Dementia Cohort; CI, confidence interval; HR, hazard ratio; MRI, magnetic resonance imaging; NACC, National Alzheimer's Coordinating Center.

### 3.2 AD versus other types of dementia

Of all dementia cases, most were diagnosed as AD (76.7% of the dementia cases in the ADC, 60.6% in NACC, and 70.3% in the Rotterdam Study). Amyloid beta 42 information was available in the ADC from CSF in 1246 (63.4%) participants and from amyloid PET imaging in 360 (18.3%) participants. Taken together, information on either measurement was available for 1214/1964 individuals of whom 387 (31.9%) were amyloid positive.

**FIGURE 2:**
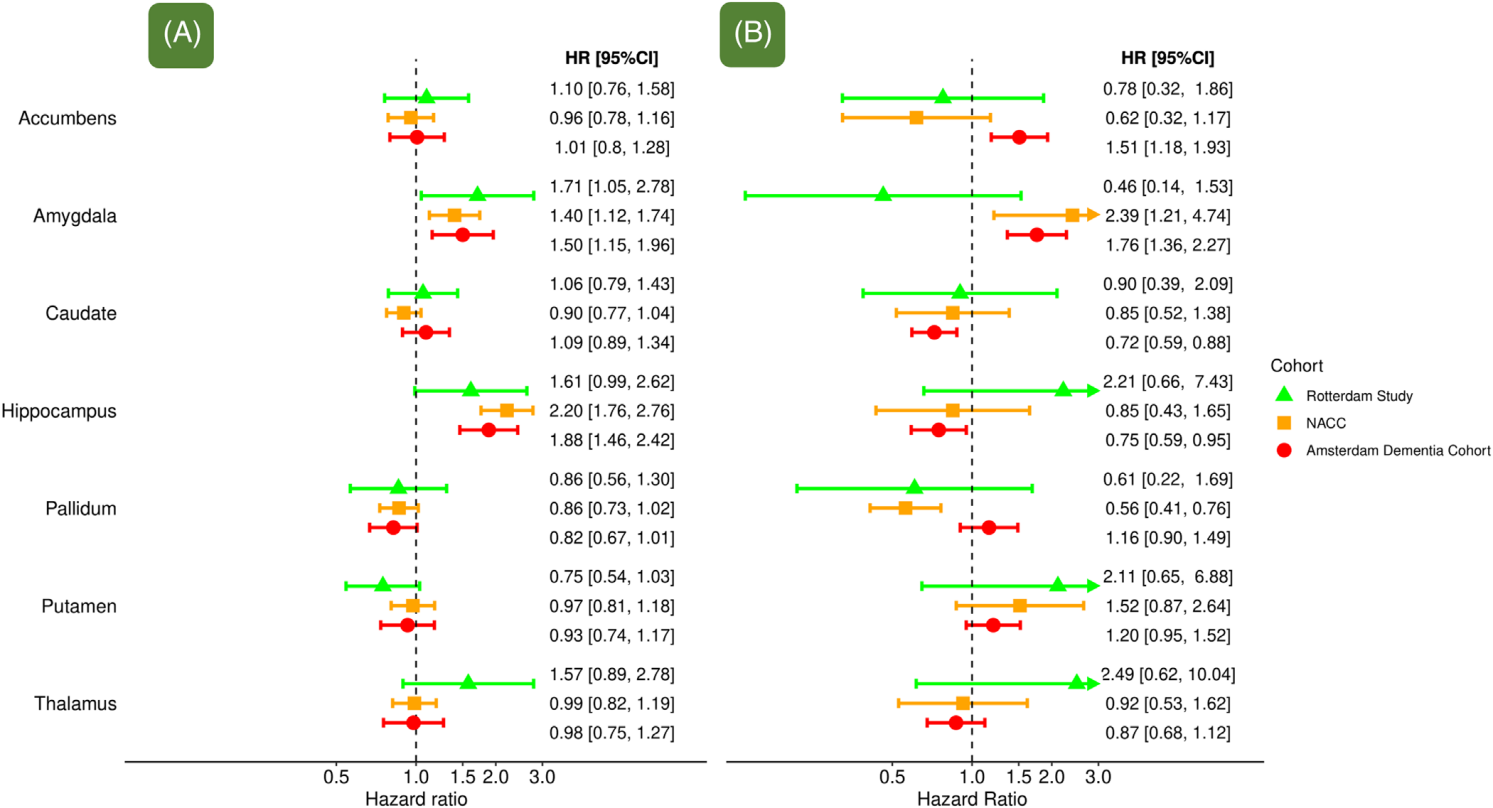
Subcortical structures and risk of dementia due to (A) Alzheimer's disease and (B) other causes of dementia. Hazard ratio per standard deviation decrease of subcortical structure volume, adjusted for age, sex, education, MRI field strength, MRI scanner, intracranial volume, cortical gray matter, and volume of all other subcortical structures. Error bars depict 95% confidence intervals. CI, confidence interval; HR, hazard ratio; MRI, magnetic resonance imaging; NACC, National Alzheimer's Coordinating Center.

In the population‐based cohort, risk estimates for the hippocampus were similar for AD and non‐AD dementia (Figure 2). In both clinic‐based cohorts, a smaller hippocampus was associated with increased risk of dementia due to AD (HR [95% CI] in NACC: 2.20 [1.76–2.76]; and in ADC: 1.88 [1.46–2.42]) but not with dementia due to other causes (HR [95% CI] in NACC: 0.85 [0.43–1.65]; and in ADC: 0.75 [0.59–0.95]; Figure 2). However, risk estimates for hippocampal volume did not differ between amyloid‐positive and amyloid‐negative patients in ADC (Table S3 in supporting information).

Lower volume of the amygdala in the clinic‐based cohorts was associated with risk of dementia due to clinical AD (HR [95% CI] in NACC: 1.40 [1.12–1.74]; and in ADC 1.50: [1.15–1.96]) as well as other dementia types (HR [95% CI] in NACC: 2.39 [1.21–4.74]; and in ADC 1.76 [1.36–2.27]). Similar patterns were seen when stratifying by amyloid positivity (Table S3). In contrast, in the population‐based cohort, amygdalar volume was associated with risk of dementia due to AD (HR [95% CI]: 1.71 [1.05–2.78]), but not other causes of dementia (HR [95% CI]: 0.46 [0.14–1.53]).

### 3.3 Disease stage

**FIGURE 3:**
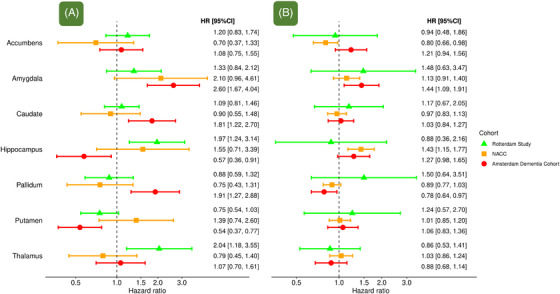
Subcortical structures and risk of dementia, stratified by clinical diagnosis of (A) subjective cognitive decline and (B) mild cognitive impairment. Hazard ratio per standard deviation decrease of subcortical structure volume, adjusted for age, sex, education, MRI field strength, MRI scanner, intracranial volume, cortical gray matter and volumes of all other subcortical structures. Error bars depict 95% confidence intervals. CI, confidence interval; HR, hazard ratio; MRI, magnetic resonance imaging; NACC, National Alzheimer's Coordinating Center.

When stratifying by SCD and MCI, no consistent major differences were observed (Figure 3). In both clinic‐based cohorts, we observed slightly higher risk estimates for the amygdala in individuals with SCD than with MCI, but this was not seen in the population‐based cohort (Figure 3). For the hippocampus, risk estimates appeared higher with SCD than with MCI in the population‐based cohort, but CIs around the MCI estimate were wide and no differences were observed in both clinic‐based cohorts (Figure 3). For the putamen, risk estimates suggested a slightly decreased dementia risk with lower volumes among individuals with SCD rather than MCI in the ADC and Rotterdam Study, but this was not observed in NACC (Figure 3).

### 3.4 Sensitivity analyses

Results from the sensitivity analysis showed no major differences in risk estimates when restricting analyses in NACC to participants who visited the research center solely for clinical evaluation (Table S4 in supporting information).

There were no consistent differences in associations between men and women. Interaction between sex and subcortical structures was statistically significant only for the hippocampus (*P* = 0.01) and thalamus (*P* = 0.02) in the ADC, but interaction went in the opposite direction in the other two cohorts (Table S5 in supporting information). Risk estimates did not change between adjustments for different MRI scanners and field strength through either neuroCombat harmonization of the volumes or through adding them as covariates in the Cox model (Table S6 in supporting information).

## 4 DISCUSSION

In this study of 7076 participants with SCD and MCI from three large cohorts, we found that hippocampal and amygdalar volumes are consistently associated with a 5‐year risk of dementia, independent of other subcortical structures and cortical gray matter volume. No consistent associations were observed with other subcortical gray matter structures.

Our analysis across cohorts robustly shows that volumes of the amygdala as well as the hippocampus are related to the progression from SCD and MCI to dementia. While the hippocampus is widely acknowledged as a marker for the risk of dementia,^29^, ^30^, ^31^, ^32^ the role of the amygdala in progression to all‐cause dementia was less established. A prior analysis among 511 participants of the Rotterdam Study showed that manual segmentations of the amygdala were associated with all‐cause dementia risk, while another clinic‐based study suggested smaller amygdala volume increases the short‐term,^33^ 1‐year risk of progression to AD dementia.^34^ These results align with a more recent multi‐cohort analysis, which demonstrated a significant association between amygdalar or hippocampal volume and the risk of AD dementia.^32^ Our study supports the notion that prognostic value of hippocampal and amygdalar volume extend to 5 years of follow‐up. Hippocampal volume appeared more strongly associated with AD dementia than with non‐AD dementia, but this was driven by the clinic‐based cohorts, in which use of hippocampal volume or visual rating scales for diagnosing AD caused some circularity and may have influenced the outcome assessment for the hippocampus. Notably, when stratifying by amyloid status, no clear differences in risk of dementia were observed between amyloid‐positive and amyloid‐negative individuals for hippocampal volume.

None of the other subcortical gray matter structures showed a clear association with risk of dementia across cohorts. On the basis of prior studies, we had hypothesized a more profound role of the thalamus, which has an important relay function between other subcortical structures. Among cognitively healthy individuals from the Rotterdam Study sample, thalamic volume was strongly associated with dementia risk.^7^ Lower volumes of the thalamus are observed during the early stage of MCI^10^, ^35^ and seem to remain stable from late MCI to AD dementia.^35^ This may explain why we did not observe significant associations between thalamic volume and dementia risk in our current study of mostly later‐stage MCI.^36^ Remarkably, the observed associations in the current study were thus confined to structures that are part of the limbic system.^37^ This may reflect involvement of the limbic system in AD, but perhaps more likely reflects contributions of limbic‐predominant age‐related TDP‐43 encephalopathy (LATE) to dementia risk. Misclassification of LATE as AD happens frequently in daily clinical practice, due to the similarity in symptoms and the absence of LATE‐specific diagnostic markers in vivo.^38^ Further research is needed to link our clinical observations to underlying pathologies, measured *post mortem* or—ideally—in vivo capitalizing on development of liquid or imaging biomarkers. Advances in imaging resolution and processing may in this regard lead to more fine‐grained observations of the hippocampus and amygdala, as well as other smaller structures that may currently not be captured in sufficient detail.

Our findings support further consideration of the amygdalar volumes for clinical decision making, in addition to an assessment of the hippocampus. Volumetric segmentations increasingly allow for incorporation of the amygdala in diagnostic and prognostic tools. As visual rating scales for medial temporal lobe atrophy may be more easily applied in clinics than volumetric assessment of the hippocampus, proper comparison of these two assessment methods is needed to determine possible added value of hippocampal volumetry. In any case, our findings imply that future research using volumetrics should go beyond the hippocampus alone, to evaluate the joint role of the amygdala and hippocampus for dementia prognosis and prediction. Moreover, such studies could explore whether repeated measurements of subcortical volumes over time could improve dementia predictive accuracy. Additionally, investigating the sequence of deterioration in various subcortical structures is important for understanding their prognostic value throughout the pre‐clinical disease trajectory.

Our study is strengthened by the inclusion of three large cohorts, including both memory clinic‐based and community‐based individuals, with comprehensive information on brain imaging and cognitive status. It is also important to consider some methodological limitations when interpreting our findings. First, different versions of FreeSurfer (i.e., 6.0 and 7.0) were used for volumetric segmentation in the included cohorts, which may have led to heterogeneity between cohorts. However, intraindividual correlation between FreeSurfer 6.0 and 7.0 segmentations of subcortical structures was high in a subsample of the Rotterdam Study (intraclass correlation ranging from 0.76 to 0.97). Second, variation in scanners, including in MRI field strength, may have induced measurement error as shown in previous research,^39^ despite adjustment in multivariable models. Yet, risk estimates were similar when restricting analyses to the 3.0T scans in the clinic‐based cohorts (Table S7 in supporting information). Third, the low number of dementia cases not attributed to AD and the relatively low number of individuals with MCI in the community‐based sample resulted in less precise estimates for these analyses. In addition, due to the non‐collapsibility properties of hazard ratios,^40^ some findings in the stratified analyses are difficult to compare directly to the primary analysis. Fifth, misclassification bias might have been introduced due to the absence of amyloid biomarker data for definite diagnosis of AD in most participants. Sixth, differential attrition may have induced selection bias, most likely attenuating findings to the null if non‐response was related to worse cognitive status. Finally, we did not have continuous follow‐up data until death for dementia of participants in NACC and ADC, precluding computation of absolute risks using competing risk modeling.

In conclusion, hippocampal and amygdalar volume are consistently associated with dementia risk in individuals with SCD or MCI, which holds the potential for personalized prognosis. Other subcortical gray matter structures showed no consistent associations with dementia risk.

## CONFLICT OF INTEREST STATEMENT

Wiesje van der Flier has been an invited speaker at Biogen MA Inc, Danone, Eisai, WebMD Neurology (Medscape), NovoNordisk, Springer Healthcare, European Brain Council. All funding is paid to her institution. Wiesje van der Flier is consultant to Oxford Health Policy Forum CIC, Roche, Biogen MA Inc, and Eisai. All funding is paid to her institution. Wiesje van der Flier participated on advisory boards of Biogen MA Inc, Roche, and Eli Lilly. Wiesje van der Flier is member of the steering committee of EVOKE/EVOKE+ (NovoNordisk). All funding is paid to her institution. Wiesje van der Flier is member of the steering committee of PAVE, and Think Brain Health. Wiesje van der Flier was associate editor of *Alzheimer, Research & Therapy* in 2020/2021. Wiesje van der Flier is associate editor at *Brain*. Author disclosures are available in the supporting information.

## CONSENT STATEMENT

All studies were approved by the relevant institutional review boards, and written informed consent was obtained from all participants.

## Supporting information



Supporting information

Supporting information
