# Supplementary material for: Subcortical gray matter volumes and 5‐year dementia risk in individuals with subjective cognitive decline or mild cognitive impairment: A multi‐cohort analysis
Source: Alzheimers Dement. 2025 Jul 8;21(7):e70413. doi: 10.1002/alz.70413 (PMC12238309; doi:10.1002/alz.70413)
Supplement: Supplementary file 1 — Supporting information [file ALZ-21-e70413-s001.docx]

**Supplemental Material:** Subcortical grey matter volumes and 5-year dementia risk in individuals with subjective cognitive decline or mild cognitive impairment: a multi-cohort analysis

Mathijs T. Rosbergen^1,2^, MSc; Pieter van der Veere^4,5,6^, MD; Jacqueline J. Claus^1,2^, MD; Tavia E. Evans^2,3^, PhD; Vikram Venkatraghavan^4,6^, PhD; Frederik Barkhof^7,8^, PhD; Argonde C. van Harten^6^, PhD; M. Arfan Ikram^1^, PhD; Wiesje M. van der Flier^4,5,6^, PhD; Meike W. Vernooij^1,2^, PhD; Frank J. Wolters^1,2^, PhD;

1. Department of Epidemiology, Erasmus MC – University Medical Center Rotterdam, PO Box 2040, Rotterdam 3000CA, the Netherlands

2. Department of Radiology & Nuclear Medicine and Alzheimer Centre, Erasmus MC – University Medical Center Rotterdam, PO Box 2040, Rotterdam 3000CA, the Netherlands

3. Department of Clinical Genetics, Erasmus MC – University Medical Center, PO Box 2040, Rotterdam 3000CA, the Netherlands

4. Amsterdam Neuroscience, Neurodegeneration, PO box 22660, 1100 DD Amsterdam Zuidoost, the Netherlands

5. Department of Epidemiology and Data Science, Amsterdam UMC location Vrije Universiteit Amsterdam, PO Box 7057, 1007 MB Amsterdam, the Netherlands

6. Alzheimer Center Amsterdam, Neurology, Vrije Universiteit Amsterdam, Amsterdam UMC location VUMC, PO Box 7057, 1007 MB Amsterdam, the Netherlands

7. Department of Radiology and Nuclear Medicine, Amsterdam Neuroscience, Vrije Universiteit Amsterdam, PO Box 7057, 1007 MB Amsterdam, the Netherlands

8. Dementia Research Centre, UCL Queen Square Institute of Neurology, University College London, PO Box 16, National Hospital for Neurology and Neurosurgery, Queen Square, London, WC1N 3BG, United Kingdom

Corresponding Author:

Mathijs T Rosbergen, MSc

Department of Epidemiology & Department of Radiology, Erasmus MC

PO Box 2040, Rotterdam 3000CA, the Netherlands

Email: m.rosbergen@erasmusmc.nl

**Table A1.**

Title: Incidence of dementia for each year follow-up.

| **Time point** | **Amsterdam Dementia Cohort** | |  | **NACC** | |  | **Rotterdam Study** | |  |
| --- | --- | --- | --- | --- | --- | --- | --- | --- | --- |
|  | **At risk** | **Cumulative dementia** | **New dementia** | **At risk** | **Cumulative dementia** | **New dementia** | **At risk** | **Cumulative dementia** | **New dementia** |
| Baseline | 1964 | 0 | - | 2207 | 0 | - | 2905 | 0 | - |
| Year 1 | 1122 | 33 | 33 | 1709 | 57 | 57 | 2873 | 7 | 7 |
| Year 2 | 857 | 104 | 71 | 1487 | 139 | 82 | 2811 | 19 | 12 |
| Year 3 | 640 | 180 | 76 | 1240 | 215 | 76 | 2738 | 39 | 20 |
| Year 4 | 478 | 226 | 46 | 996 | 265 | 50 | 2662 | 60 | 21 |
| Year 5 | 355 | 249 | 23 | 821 | 292 | 27 | 2553 | 81 | 21 |

Numbers include time till last assessment, which may have underestimated the speed of progression in some participants who for example missed their 2-year examination before being diagnosed with dementia at the year-3 visit.

**Table A2.**

Title: Predictive power when adding hippocampal volume and amygdalar volume.

| Model | **C-statistic**  **[95%CI]**  **NACC** | **C-statistic**  **[95%CI]**  **ADC** | **C-statistic**  **[95%CI]**  **Rotterdam Study** |
| --- | --- | --- | --- |
| Age + sex + education | 0.703  [0.675-0.731] | 0.635  [0.599-0.670] | 0.847  [0.817-0.877] |
| Age + sex + education + hippocampus | 0.790  [0.765-0.815] | 0.745  [0.715-0.775] | 0.870  [0.842-0.898] |
| Age + sex + education + hippocampus + amygdala | 0.800  [0.776-0.824] | 0.761  [0.731-0.790] | 0.870  [0.840-0.901] |

Abbreviations: ADC=Amsterdam Dementia Cohort, NACC=National Alzheimer’s Coordinating Center, HR=hazard ratio, CI=confidence interval

**Table** **A3**.

Title: Subcortical structures and risk of dementia, stratified by amyloid positivity

Legend: Results from a subsample of the Amsterdam Dementia Cohort.

| **Subcortical structure** | **Amyloid status** | **N (%)** | **HR [95%CI]** | **P interaction** |
| --- | --- | --- | --- | --- |
| Accumbens | Positive | 387 (31.9) | 1.24 [0.88-1.74] | 0.29 |
|  | Negative | 827 (68.1) | 1.55 [0.89-2.69] |  |
| Amygdala | Positive | 387 (31.9) | 1.56 [1.10-2.21] | 0.12 |
|  | Negative | 827 (68.1) | 2.58 [1.42-4.71] |  |
| Caudate | Positive | 387 (31.9) | 0.92 [0.72-1.17] | 0.41 |
|  | Negative | 827 (68.1) | 0.96 [0.62-1.48] |  |
| Hippocampus | Positive | 387 (31.9) | 1.26 [0.91-1.74] | 0.32 |
|  | Negative | 827 (68.1) | 1.17 [0.64-2.13] |  |
| Pallidum | Positive | 387 (31.9) | 0.74 [0.53-1.02] | 0.78 |
|  | Negative | 827 (68.1) | 0.81 [0.49-1.36] |  |
| Putamen | Positive | 387 (31.9) | 1.12 [0.81-1.55] | 0.94 |
|  | Negative | 827 (68.1) | 1.05 [0.62-1.75] |  |
| Thalamus | Positive | 387 (31.9) | 1.04 [0.75-1.45] | 0.57 |
|  | Negative | 827 (68.1) | 0.52 [0.25-1.07] |  |

Hazard ratio per standard deviation increase of volume. P interaction shows p-value for interaction between volume of subcortical structures and amyloid status. Abbreviations: HR=hazard ratio, CI=confidence interval, NACC=National Alzheimer’s Coordinating Center.

**Table A4.**

Title: Subcortical structures and risk of dementia for participants in the National Alzheimer’s Coordinating Center cohort.

Legend: Results from a Cox regression model for all participants in the National Alzheimer’s Coordinating Center cohort and a subset of participants who visited the research center solely for a clinical evaluation.

|  | **NACC,**  **all participants  HR [95%CI]**  **N=2207** | **NACC,**  **participants who attended research center for clinical evaluation HR [95%CI]**  **N=507** |
| --- | --- | --- |
| Accumbens | 0.93 [0.78-1.12] | 1.08 [0.75-1.57] |
| Amygdala | 1.46 [1.19-1.79] | 1.45 [1.00-2.08] |
| Caudate | 0.92 [0.80-1.06] | 1.07 [0.78-1.46] |
| Hippocampus | 1.92 [1.56-2.37] | 1.51 [1.05-2.17] |
| Pallidum | 0.80 [0.69-0.93] | 0.76 [0.59-0.96] |
| Putamen | 1.04 [0.87-1.24] | 0.78 [0.56-1.08] |
| Thalamus | 1.02 [0.86-1.22] | 0.95 [0.67-1.34] |

Hazard ratios adjusted for MRI scanner (settings), intracranial volume, age, sex, education, other subcortical structure volumes and cortical grey matter volume.

Abbreviations: NACC=National Alzheimer’s Coordinating Center, HR=hazard ratio, CI=confidence interval

**Table** **A5.**

Title: Subcortical structures and the risk of dementia stratified by sex.

| **Subcortical structure** | **Sex** | **Rotterdam Study** | | **NACC** | | **Amsterdam Dementia Cohort** | |
| --- | --- | --- | --- | --- | --- | --- | --- |
|  |  | HR [95%CI] | P interaction | HR [95%CI] | P interaction | HR [95%CI] | P interaction |
| Accumbens | Female | 1.18  [0.78-1.79] | 0.81 | 0.98  [0.74-1.31] | 0.83 | 1.10  [0.76-1.59] | 0.86 |
|  | Male | 1.17  [0.71-1.93] |  | 0.96  [0.75-1.23] |  | 1.23  [0.94-1.60] |  |
| Amygdala | Female | 1.76  [1.02-3.03] | 0.78 | 1.94  [1.41-2.67] | 0.77 | 1.33  [0.87-2.04] | 0.59 |
|  | Male | 1.12  [0.60-2.09] |  | 1.23  [0.94-1.62] |  | 1.79  [1.32-2.43] |  |
| Caudate | Female | 0.92  [0.66-1.30] | 0.25 | 0.99  [0.80-1.21] | 0.79 | 0.96  [0.66-1.38] | 0.33 |
|  | Male | 1.43  [0.95-2.16] |  | 0.88  [0.72-1.07] |  | 1.04  [0.85-1.27] |  |
| Hippocampus | Female | 1.34  [0.79-2.28] | 0.55 | 1.74  [1.27-2.37] | 0.49 | 2.28  [1.52-3.43] | 0.01 |
|  | Male | 2.19  [1.13-4.24] |  | 1.99  [1.49-2.66] |  | 1.23  [0.94-1.65] |  |
| Pallidum | Female | 0.94  [0.59-1.48] | 0.36 | 0.74  [0.59-0.93] | 0.19 | 0.92  [0.67-1.27] | 0.44 |
|  | Male | 1.18  [0.64-2.18] |  | 0.83  [0.68-1.01] |  | 0.90  [0.72-1.14] |  |
| Putamen | Female | 0.98  [0.67-1.42] | 0.86 | 0.89  [0.68-1.16] | 0.15 | 0.83  [0.55-1.27] | 0.55 |
|  | Male | 0.58  [0.32-1.04] |  | 1.14  [0.89-1.45] |  | 0.98  [0.78-1.23] |  |
| Thalamus | Female | 0.99  [0.53-1.84] | 0.11 | 0.88  [0.68-1.13] | 0.08 | 1.31  [0.88-1.94] | 0.02 |
|  | Male | 3.07  [1.43-6.6] |  | 1.22  [0.93-1.60] |  | 0.78  [0.60-1.02] |  |

Hazard ratio per standard deviation increase of volume. P interaction shows p-value for interaction between volume of subcortical structures and sex. Abbreviations: HR = hazard ratio, CI = confidence interval, NACC=National Alzheimer’s Coordinating Center.

**Table A6.**

Title: Subcortical structures and risk of dementia for different types of adjustment for the variety of MRI scanners.

|  | **NACC** | | **Amsterdam Dementia Cohort** | |
| --- | --- | --- | --- | --- |
|  | **NeuroCombat harmonization HR [95%CI]** | **Adjusting in  Cox model HR [95%CI]** | **NeuroCombat harmonization  HR [95%CI]** | **Adjusting in  Cox model**  **HR [95%CI]** |
| Accumbens | 0.94 [0.79-1.13] | 0.93 [0.78-1.12] | 1.07 [0.88-1.31] | 1.11 [0.90-1.35] |
| Amygdala | 1.46 [1.19-1.79] | 1.46 [1.19-1.79] | 1.57 [1.25-1.96] | 1.59 [1.26-2.01] |
| Caudate | 0.92 [0.80-1.05] | 0.92 [0.80-1.06] | 1.08 [0.91-1.28] | 1.00 [0.85-1.19] |
| Hippocampus | 1.95 [1.58-2.40] | 1.92 [1.56-2.37] | 1.53 [1.22-1.93] | 1.55 [1.25-1.93] |
| Pallidum | 0.78 [0.68-0.90] | 0.80 [0.69-0.93] | 0.88 [0.73-1.07] | 0.89 [0.75-1.07] |
| Putamen | 1.04 [0.88-1.25] | 1.04 [0.86-1.22] | 0.95 [0.78-1.16] | 0.97 [0.80-1.18] |
| Thalamus | 1.05 [0.88-1.24] | 0.93 [0.75-1.17] | 0.87 [0.70-1.09] | 0.93 [0.75-1.17] |

Hazard ratios adjusted for intracranial volume, age, sex, education, other subcortical structure volumes and cortical grey matter volume. Adjustment for MRI scanner was performed by either as covariate in the Cox proportional hazards model or by harmonizing imaging measurements using NeuroCombat harmonization.

Abbreviations: NACC=National Alzheimer’s Coordinating Center, HR=hazard ratio, CI=confidence interval, MRI=magnetic resonance imaging

**Table A7.**

Title: Subcortical structures and risk of dementia for different MRI field strengths.

Legend: Results from a Cox regression model for all participants in the National Alzheimer’s Coordinating Center cohort and the Amsterdam Dementia Cohort and a subset of participants who underwent brain MRI with 3.0T field strength.

|  | **NACC** | | **Amsterdam Dementia Cohort** | |
| --- | --- | --- | --- | --- |
|  | **All participants  HR [95%CI]**  **N=2207** | **Participants with  3.0T MRI HR [95%CI]**  **N=1316** | **All participants  HR [95%CI]**  **N=1964** | **Participants with 3.0T MRI HR [95%CI]**  **N=1410** |
| Accumbens | 0.93 [0.78-1.12] | 0.87 [0.67-1.13] | 1.11 [0.90-1.35] | 1.15 [0.90-1.46] |
| Amygdala | 1.46 [1.19-1.79] | 1.57 [1.20-2.05] | 1.59 [1.26-2.01] | 1.84 [1.38-2.44] |
| Caudate | 0.92 [0.80-1.06] | 0.96 [0.79-1.17] | 1.00 [0.85-1.19] | 1.11 [0.91-1.37] |
| Hippocampus | 1.92 [1.56-2.37] | 1.94 [1.47-2.56] | 1.55 [1.25-1.93] | 1.51 [1.16-1.96] |
| Pallidum | 0.80 [0.69-0.93] | 0.80 [0.66-0.97] | 0.89 [0.75-1.07] | 0.88 [0.70-1.10] |
| Putamen | 1.04 [0.87-1.24] | 1.02 [0.79-1.33] | 0.97 [0.80-1.18] | 0.98 [0.77-1.24] |
| Thalamus | 1.02 [0.86-1.22] | 1.19 [0.91-1.54] | 0.93 [0.75-1.17] | 0.79 [0.61-1.03] |

Hazard ratios adjusted for MRI scanner, intracranial volume, age, sex, education, other subcortical structure volumes and cortical grey matter volume.

Abbreviations: NACC=National Alzheimer’s Coordinating Center, HR=hazard ratio, CI=confidence interval, MRI=magnetic resonance imaging
